# CRISPR-based next-generation molecular diagnostics for bone infection

**DOI:** 10.3389/fcell.2026.1876644

**Published:** 2026-06-17

**Authors:** Ju Chen, Hao Tan, Yu Guo, Zhihui Jiang, Jiupan Zhu, Han Wang, Shiqi Xiao, Dianxiang Lu, Sheng Ding, Jin Yang

**Affiliations:** Clinical Medical College and Affiliated Hospital, Chengdu University, Chengdu, China

**Keywords:** AMR (antimicrobial resistance), bone infection, CRISPR-diagnosics, osteomyelitis (OM), POCT (point-of-care testing)

## Abstract

Bone and joint infections, notably osteomyelitis and periprosthetic joint infections, present a profound clinical challenge characterized by severe morbidity, prolonged hospitalizations, and substantial healthcare costs. Effective management is persistently hindered by diagnostic uncertainty. Conventional microbiological cultures yield false-negative results in up to 50% of cases, largely due to prior antibiotic exposure, biofilm formation, and fastidious organisms. Concurrently, the escalating prevalence of antimicrobial resistance (AMR) among orthopedic pathogens demands rapid and highly accurate diagnostic alternatives. Clustered Regularly Interspaced Short Palindromic Repeats (CRISPR) and CRISPR-associated (Cas) technologies have recently emerged as robust nucleic acid detection platforms. These systems offer attomolar sensitivity and single-nucleotide specificity while remaining compatible with portable, instrument-free readout formats. In this review, we critically evaluate the current diagnostic landscape of bone infections, highlighting the limitations of conventional and early molecular methods. We specifically examine how the distinct mechanistic properties of varied Cas effectors (Cas12, Cas13, Cas14, and CasΦ) can transform clinical diagnostics through single-nucleotide polymorphism (SNP) discrimination, RNA-based viability assessment, and PAM-independent detection. Furthermore, we explore the application of these features in stratifying pathogens within culture-negative and polymicrobial infections, directly identifying AMR genes for targeted therapy, and facilitating point-of-care testing in intraoperative environments. Finally, we assess the primary barriers to clinical translation, namely, complex sample preprocessing in bone matrices, the lack of standardized diagnostic thresholds, and the need for prospective studies demonstrating improved patient outcomes, ultimately outlining essential research priorities to transition CRISPR diagnostics from laboratory proof-of-concept to routine orthopedic practice.

## 1 Introduction

Bone infections, including acute osteomyelitis, septic arthritis, and periprosthetic joint infections, represent a substantial clinical burden characterized by prolonged hospitalizations. Diagnostic delays and underlying healthcare disparities further exacerbate morbidity early in the disease course (Campbell et al., 2023). Chronic presentations severely worsen patient outcomes; for instance, the presence of osteomyelitis in diabetic foot ulcers substantially increases the risk of lower extremity amputation and escalates direct healthcare costs (Zhang et al., 2023; Lew et al., 2025). Furthermore, concurrent extremity osteomyelitis significantly elevates mortality rates in patients with systemic infections (Kim et al., 2023). Microbiological complexity compounds these clinical challenges. *Staphylococcus aureus*, including methicillin-resistant strains, predominates in pediatric osteomyelitis (Chen et al., 2024). Conversely, adult and orthopedic device infections frequently involve polymicrobial communities and biofilm formations (Chauvelot et al., 2020). The escalating prevalence of antimicrobial resistance severely restricts effective therapeutic options (Zhao et al., 2019; Elsayed et al., 2024; Zhang et al., 2024). This convergence of biofilm persistence, polymicrobial etiology, and resistance renders empirical broad-spectrum therapy frequently ineffective, necessitating rapid and precise pathogen identification to prevent recurrent infection and tissue destruction.

Current diagnostic modalities remain inadequate for these challenges. Conventional cultures are time-consuming and prone to false-negative results following prior antibiotic exposure, whereas systemic inflammatory markers lack sufficient specificity. This diagnostic ambiguity delays targeted antimicrobial therapy and contributes to avoidable tissue loss. Resolving this dilemma requires rapid, molecular pathogen detection. Clustered Regularly Interspaced Short Palindromic Repeats (CRISPR) technologies have recently emerged as a robust diagnostic platform. This review examines the diagnostic limitations in bone infections, the mechanisms of CRISPR nucleic acid detection, and clinical applications in pathogen identification, antimicrobial resistance profiling, and point-of-care testing. We then assess how CRISPR technologies could change the clinical management of bone infections.

## 2 The diagnostic dilemma of bone infection

The diagnosis of bone infection remains a persistent clinical challenge primarily due to the limitations of conventional microbiological culture. Culture-negative results are prevalent, often exacerbated by prior antibiotic administration (Shin et al., 2020; Chen et al., 2020). In pediatric osteoarticular infections, pathogen detection rates remain suboptimal across diverse clinical specimens (Shin et al., 2020). When cultivation is successful, the prolonged turnaround time inevitably delays targeted treatment and necessitates extended reliance on empirical broad-spectrum antibiotics. Furthermore, standard inflammatory markers lack reliable diagnostic precision. Imaging modalities, while sensitive to local inflammation, possess insufficient specificity to differentiate active infections from non-infectious mimics such as chronic non-bacterial osteomyelitis (D’Angelo et al., 2021).

Diagnostic yields are additionally compromised by complex pathogen biology and the local bone microenvironment. Fastidious organisms like *Kingella kingae*, alongside atypical pathogens such as *Mycobacterium tuberculosis* and fungi, frequently evade standard culture and require specialized, time-consuming protocols (Santos et al., 2020). Biofilm formation on osseous tissues and orthopedic implants further obscures pathogen detection while drastically elevating antimicrobial tolerance (Chauvelot et al., 2020). Although advanced molecular techniques like metagenomic next-generation sequencing demonstrate significantly higher diagnostic sensitivity than standard culture (Xiao et al., 2025), their clinical application is constrained by substantial costs and the necessity for centralized laboratory infrastructure. Furthermore, interpreting these diagnostics is complicated by the lack of universally standardized criteria, as current composite clinical guidelines may overlook early or subacute presentations (Trapero et al., 2021). Ultimately, the synergistic challenges of insensitive cultures, non-specific biomarkers, ambiguous imaging, and biofilm interference perpetuate a critical diagnostic gap that emerging molecular platforms are positioned to resolve.

## 3 The rise of CRISPR-Dx

**FIGURE 1:**
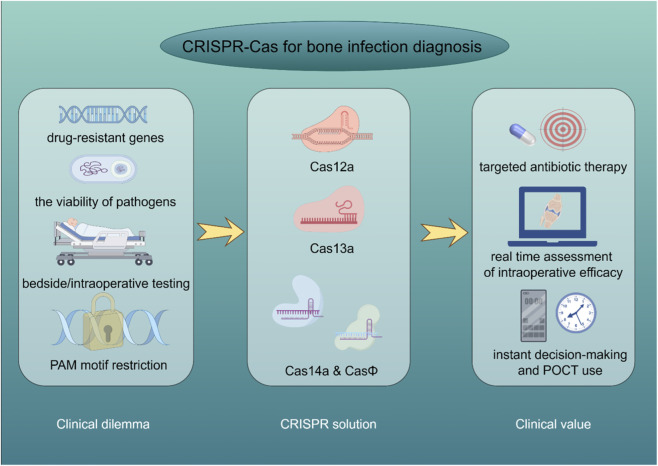
Overview of CRISPR-Cas effectors for resolving diagnostic dilemmas in bone infections. The left panel depicts four major clinical and analytical challenges: The central panel illustrates how distinct Cas effector enzymes address these challenges: Cas12a enables precise SNP discrimination for AMR gene identification; Cas13a targets bacterial mRNA to assess viability; miniature Cas14 and CasΦ offer PAM-independent detection suitable for portable platforms. The right panel shows the resulting clinical utilities, including targeted antibiotic therapy, real-time monitoring of treatment response, and decentralized point-of-care testing.

CRISPR-Cas diagnostic platforms leverage programmable nucleic acid recognition coupled with the collateral cleavage activity of specific Cas effectors (Chen et al., 2018; Gootenberg et al., 2018). Upon target binding via a complementary guide RNA (crRNA), these effectors undergo conformational changes to indiscriminately cleave surrounding single-stranded nucleic acids. By incorporating labeled reporter oligonucleotides, this collateral activity generates measurable fluorescence or colorimetric signals proportional to the target concentration (Chen et al., 2018). For bone infection diagnostics, a key advantage is isothermal signal amplification: single-molecule sensitivity without thermocycling (Chen et al., 2018; Gootenberg et al., 2018). The following subsections examine how distinct Cas effectors address specific diagnostic challenges in orthopedic infections (Figure 1).

### 3.1 Cas12

Cas12a is an RNA-guided endonuclease that exhibits collateral single-stranded DNA cleavage upon recognizing a complementary double-stranded DNA target (Chen et al., 2018). Its activation requires a protospacer adjacent motif (PAM), which serves as an initial specificity filter (Chen et al., 2018). Structural studies confirm that target binding induces specific conformational changes necessary for catalytic activation (Garcia-Doval and Jinek, 2017). Strict complementarity in the crRNA seed region dictates this activation, enabling the precise discrimination of single-nucleotide polymorphisms (SNPs) (Tambe et al., 2018). Clinically, this specificity allows assays to differentiate antimicrobial resistance alleles, effectively distinguishing methicillin-resistant *Staphylococcus aureus* (MRSA) from susceptible strains without requiring whole-genome sequencing (Du and Xiu, 2024). The DETECTR platform, coupling Cas12a with isothermal amplification, established the clinical viability of highly specific DNA detection (Chen et al., 2018). Subsequent adaptations have successfully detected resistance genes in orthopedic clinical specimens with exceptional sensitivity (Du and Xiu, 2024; Harb et al., 2023; Jiang et al., 2025). Furthermore, multiplexed platforms can simultaneously identify pathogens and diverse antimicrobial resistance determinants (Zhao et al., 2025). The programmability of crRNA and compatibility with multiplexed isothermal amplification position Cas12a as an optimal effector for definitive species identification and resistance genotyping from clinical tissue extracts.

### 3.2 Cas13

Cas13a is an RNA-guided RNA endonuclease that triggers collateral cleavage of single-stranded RNA upon target recognition (Abudayyeh et al., 2017). This RNA-targeting capability provides distinct clinical advantages. First, the natural abundance of ribosomal and messenger RNA facilitates inherent signal amplification (Wang et al., 2025). Second, RNA detection allows for the functional profiling of transcribed virulence factors and resistance determinants (Chauvelot et al., 2020). Finally, because bacterial mRNA degrades rapidly, Cas13a detection serves as a reliable biochemical correlate of pathogen viability, which is highly advantageous for post-treatment monitoring where residual DNA might otherwise cause false-positive results (Gootenberg et al., 2018; Wang et al., 2025). The SHERLOCK assay pairs Cas13a with isothermal amplification to achieve attomolar sensitivity and rapid turnaround times (Gootenberg et al., 2018). However, translating this viability assessment to clinical bone specimens presents practical challenges. Bacterial mRNA is inherently labile, with a half-life of minutes to hours, and degrades rapidly during the aggressive physical processing often required for bone samples, such as cryogenic grinding, probe sonication, or bead beating. These procedures generate frictional heat and mechanically release endogenous RNases from lysed cells and tissue debris, risking false-negative results unless strict cold-chain maintenance and immediate RNA stabilization are implemented. To mitigate degradation risks, future assay configurations should integrate on-sample RNA preservation buffers (e.g., RNAlater), one-pot lysis-and-detection systems that minimize handling steps, or chemical lysis protocols that simultaneously inactivate RNases. Without such precautions, Cas13a-based viability assessment may underestimate viable pathogen loads in clinical bone biopsies. Clinical applications have successfully identified bacterial RNA directly from primary specimens (Wang et al., 2025). Advanced iterations incorporate auxiliary enzymes to enable the quantitative measurement of pathogen loads (Gootenberg et al., 2018). Utilizing a protospacer flanking sequence rather than a PAM expands targetable genomic regions (Meeske and Marraffini, 2018). Cas13a activation relies on specific Higher Eukaryotes and Prokaryotes Nucleotide-binding domains, with proximal mismatches effectively abrogating cleavage activity (Tambe et al., 2018; Zhang et al., 2018). Its capacity for viability assessment, quantitative output, and direct sample testing renders Cas13a highly applicable for monitoring therapeutic responses in bone infections (Myhrvold et al., 2018).

### 3.3 Cas14 and CasΦ

The physical and biochemical properties of newer Cas effectors facilitate the development of portable diagnostic systems. Cas14 is substantially smaller than conventional effectors and cleaves single-stranded DNA targets without PAM sequence restrictions (Harrington et al., 2018). Despite its size, its collateral cleavage generates robust signal amplification (Harrington et al., 2018). Diagnostic biosensors utilizing Cas14 have achieved sensitive, instrument-free nucleic acid detection with colorimetric readouts suitable for smartphone integration (Zhao et al., 2024). This versatile platform has also been expanded to detect non-nucleic acid targets (Zhou et al., 2023). In orthopedic contexts, Cas14 has successfully identified resistance genes in MRSA and vancomycin-resistant enterococci (Agha et al., 2025; Zhao et al., 2024). Additionally, CasΦ is a miniature RNA-guided DNA endonuclease that operates entirely independently of PAM constraints and maintains full catalytic activity at standard human body temperature (Liu et al., 2025). This independence eliminates sequence selection limitations, while its optimal temperature profile simplifies the thermal management required for point-of-care testing (Liu et al., 2025). This PAM independence provides two key advantages for portable diagnostic platforms: first, it simplifies guide RNA design by eliminating the need to locate a PAM sequence adjacent to the target site, thereby accelerating assay development; second, it substantially expands the accessible genomic universe, particularly benefiting targets in AT-rich pathogen genomes or regions where PAM sites are sparse—a common limitation of Cas12a-based assays. Proof-of-concept studies demonstrate the utility of CasΦ in broadly identifying bacterial ribosomal genes and specific carbapenemase determinants (Liu et al., 2025). Collectively, the compact physical footprint, broad substrate tolerance, and reduced operational requirements of Cas14 and CasΦ expand the utility of CRISPR diagnostics for decentralized orthopedic applications.

### 3.4 Comparative functional profiles and orthopedic deployment considerations

To address the complex diagnostic requirements of bone and joint infections, these four effector classes offer complementary analytical capabilities. Cas12a provides superior specificity for DNA targets and multiplexed resistance genotyping (Chen et al., 2018; Zhao et al., 2025; Du and Xiu, 2024). Cas13a delivers RNA-based functional profiling and viability assessment critical for treatment monitoring (Gootenberg et al., 2018; Wang et al., 2025). Cas14 facilitates ultra-compact, PAM-independent detection suitable for instrument-free diagnostic devices (Zhao et al., 2024; Harrington et al., 2018). CasΦ further streamlines point-of-care deployment via PAM-independent DNA detection optimized for physiological temperatures (Liu et al., 2025). Consequently, the selection of specific CRISPR effectors must be strategically aligned with the intended clinical objective, whether it involves precise species identification, resistance profiling, or viability assessment. This effector-level modularity distinguishes CRISPR diagnostics from conventional single-analyte assays, establishing a versatile toolkit capable of optimizing the orthopedic diagnostic workflow.

## 4 Clinical applications of CRISPR-based diagnostics in orthopedic infection management

**FIGURE 2:**
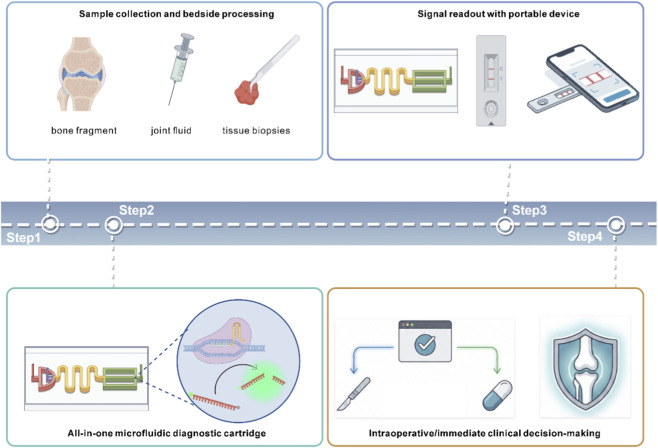
Proposed clinical workflow for the integration of CRISPR-based diagnostics into orthopedic infection management. At the first stage, bone biopsy, synovial fluid, or periprosthetic tissue specimens undergo rapid lysis and nucleic acid release using a simplified, cartridge-based extraction procedure. In the second stage, the released nucleic acids are transferred into a unified reaction chamber containing freeze-dried CRISPR reagents, where isothermal amplification and multiplexed detection occur simultaneously. The third stage shows instrument-free signal readout through a lateral flow strip or a smartphone-integrated fluorescence detector, which quantifies the signal and displays the result on a mobile application. In the final clinical decision stage, the readout directly informs intraoperative decisions on debridement adequacy and guides immediate, targeted antibiotic selection, ultimately contributing to improved patient outcomes and antimicrobial stewardship.

The clinical utility of CRISPR-based diagnostics in orthopedic infections encompasses three interconnected domains: identifying elusive pathogens, detecting antimicrobial resistance determinants to guide targeted therapy, and translating these assays into point-of-care and intraoperative platforms. Successful integration of these technologies must directly address the diagnostic gaps inherent in current clinical workflows (Figure 2).

### 4.1 Rapid pathogen identification in culture-negative and biofilm-associated infections

Pathogen identification remains severely compromised in culture-negative and biofilm-associated infections. In pediatric osteoarticular infections, standard cultivation from clinical specimens frequently yields false-negative results (Shin et al., 2020). This is primarily attributed to prior antibiotic exposure and fastidious organisms such as *Kingella kingae* (Chauvelot et al., 2020). In adult cohorts, atypical pathogens including *Mycobacterium tuberculosis* and fungi further exacerbate diagnostic delays (Santos et al., 2020). Additionally, extracellular matrices in orthopedic device-related infections obscure biofilm-embedded bacteria, shielding them from both detection and antimicrobial eradication (Chauvelot et al., 2020). Consequently, a substantial proportion of bone infections remain microbiologically undefined under conventional culture protocols (Shin et al., 2020; Chen et al., 2020).

Although metagenomic next-generation sequencing (mNGS) significantly improves detection sensitivity over standard culture (Xiao et al., 2025), its requirement for centralized infrastructure and prolonged turnaround times precludes its utility in urgent surgical settings. Adjunctive molecular diagnostics, such as host immune biomarker panels, exhibit superior sensitivity for infection detection but critically fail to identify the specific pathogen or its susceptibility profile (Shahi et al., 2016; Jenum et al., 2016). CRISPR diagnostics specifically address this limitation by providing direct, isothermal nucleic acid amplification and detection (Chen et al., 2018; Gootenberg et al., 2018). These platforms demonstrate high specificity for bone-relevant pathogens, effectively discriminating structurally similar species without cross-reactivity (Chauvelot et al., 2020; Du and Xiu, 2024). When combined with complementary intraoperative techniques like Raman spectroscopy (Lindtner et al., 2023), CRISPR-based methods facilitate a comprehensive diagnostic strategy that simultaneously captures pathogen identity and host response.

### 4.2 CRISPR-based detection of antimicrobial resistance determinants

The escalating prevalence of antimicrobial resistance in bone-infecting organisms necessitates rapid resistance profiling. High rates of methicillin and penicillin resistance among Gram-positive isolates, alongside increasing cephalosporin resistance in Gram-negative pathogens, frequently render empirical therapies ineffective (Chen et al., 2024). Targeted antimicrobial deployment requires rapid and highly sensitive diagnostic alternatives.

CRISPR platforms detect critical resistance determinants with sensitivities comparable to standard polymerase chain reaction assays while requiring minimal instrumentation. Assays utilizing Cas12a have successfully identified the mecA gene with exceptional specificity (Du and Xiu, 2024). Similarly, Cas14a demonstrates complete specificity for identifying vancomycin resistance markers (Agha et al., 2025). Furthermore, multiplexed CRISPR systems can simultaneously detect multiple resistance mutations, illustrating their adaptability for concurrent pathogen and resistance profiling in a single clinical specimen (Zhao et al., 2025).

Clinical experience with molecular screening further supports this approach. Rapid identification of resistance genes directly influences antimicrobial selection in diabetic foot infections and accurately correlates with deep tissue culture results (Harb et al., 2023). Given the substantial colonization rates of multidrug-resistant organisms in healthcare settings, preoperative molecular screening is increasingly critical before orthopedic procedures (Nucleo et al., 2018). For paucibacillary bone specimens, CRISPR technologies provide distinct advantages over conventional methods by leveraging isothermal processing and collateral cleavage to achieve superior signal amplification (Oueslati et al., 2018).

### 4.3 Translation toward point-of-care and intraoperative use

The ultimate clinical impact of CRISPR diagnostics depends on their successful deployment at the point of care. Current intraoperative diagnostics, such as frozen section histopathology, exhibit suboptimal sensitivity and specificity (Ratnavelu et al., 2016). While adjunctive tests and real-time optical tissue analysis improve intraoperative accuracy, they currently force a compromise between processing speed and comprehensive microbiological profiling (Shahi et al., 2016; Lindtner et al., 2023; Dongen et al., 2020).

CRISPR-based systems aim to eliminate this compromise by integrating nucleic acid extraction, isothermal amplification, and precise detection into unified platforms. Lateral flow assays combined with alternative readout technologies enable rapid and highly sensitive RNA detection (Joung et al., 2025). Similarly, paper-based biosensors utilizing visual colorimetric signals provide robust DNA detection suitable for decentralized environments (Zhao et al., 2024). Advancements in smartphone-integrated quantification further facilitate cost-effective and highly reproducible assay readouts (Collins et al., 2024; Yue et al., 2021). This decentralized approach has also been validated for single nucleotide polymorphism detection, offering an instrument-free strategy for resistance subtyping (Wen et al., 2021).

A key step toward truly decentralized testing is developing extraction-free protocols that bypass traditional column- or bead-based purification. Recent advances in CRISPR-Dx have demonstrated direct detection from crude clinical lysates using only brief heat treatment or alkaline lysis, followed by neutralization and direct addition to the CRISPR reaction. Although validation in bone matrices remains limited, preliminary evidence suggests that for joint fluid or bone marrow aspirates, simple dilution or the addition of chelating agents may sufficiently relieve inhibition by calcium ions or heme. For solid bone biopsies, brief probe sonication or chemical lysis in guanidine-based buffers, without subsequent purification, can release adequate nucleic acid for amplification and detection. Achieving robust extraction-free detection in these challenging samples would markedly accelerate intraoperative deployment, reduce hands-on time, and minimize contamination risks.

Translating these platforms for orthopedic applications requires rigorous validation using complex clinical matrices, including bone biopsies and periprosthetic tissues. Preliminary studies indicate that CRISPR detection maintains robust sensitivity even in the presence of bone marrow aspirates and tissue inhibitors (Harb et al., 2023). Additionally, the development of freeze-dried CRISPR reagents ensures long-term stability without cold chain logistics, facilitating deployment in resource-limited surgical settings (Rybnicky et al., 2022). The combination of sample-tolerant biochemistry, portable readouts, and lyophilized reagents makes CRISPR diagnostics a compelling tool for intraoperative use. Future clinical studies should compare these platforms against composite reference standards to determine whether they can improve surgical decision-making and patient outcomes.

## 5 Discussion

Whether CRISPR-based diagnostics translate into orthopedic practice will depend on more than analytical sensitivity or specificity. The real question is whether these tools can fit into clinical workflows in ways that change what clinicians do. Current evidence shows that CRISPR assays detect bone pathogens and resistance markers with accuracy rivaling or exceeding culture. But the gap between technical performance and clinical adoption remains large. One dimension of this gap is the lack of standardized processing protocols: bone biopsies, synovial fluid, and periprosthetic tissue each have distinct inhibitory profiles. Bone matrix contains several inhibitors that interfere with CRISPR-Cas collateral cleavage. Calcium ions, abundant in bone mineral, can chelate reaction components or disrupt nucleic acid binding and Cas catalysis. Collagen, the dominant organic matrix protein, nonspecifically adsorbs nucleic acids and polymerases, lowering reaction efficiency. Heme from residual blood in bone biopsies potently inhibits DNA polymerases and fluorescent reporters, weakening both isothermal amplification and signal readout. Mitigating these matrix-derived inhibitors will require systematic optimization of lysis buffers and reaction chemistries. Introducing chelating agents, carrier proteins, or dilution strategies can neutralize or remove interfering substances without compromising assay speed. A second dimension is multiplexing: chronic orthopedic infections are often polymicrobial and multidrug-resistant, so diagnostic panels need to report species identity and resistance determinants simultaneously. This capability remains largely at the proof-of-concept stage for CRISPR platforms in orthopedics.

The greatest value of CRISPR diagnostics may not be in replacing existing molecular methods but in moving diagnostic capacity closer to the patient. Because signal amplification is biochemical rather than thermal, these assays work with minimal instrumentation and can be read by a smartphone. This makes it feasible to bring pathogen identification and resistance profiling into outpatient clinics, emergency departments, and intraoperative settings, where the results could directly guide debridement and antibiotic choice. Realizing this vision will require clinical trials that measure more than test accuracy: the proportion of patients receiving targeted therapy within the first surgical window, the reduction in empiric broad-spectrum antibiotic days, and the impact on infection recurrence and limb salvage. Without this evidence, CRISPR diagnostics risk becoming an elegant technical solution to a problem that has not yet been shown to benefit from molecular precision over clinical judgment.
